# Lebrikizumab for adolescent atopic dermatitis: 24-week real-world outcomes from the Czech BIOREP registry

**DOI:** 10.1016/j.jdin.2026.01.009

**Published:** 2026-02-01

**Authors:** Filip Rob, Blanka Pinková, Petra Gkalpakioti, Jiřina Bartoňová, Jiří Horažďovský, Jan Říčař, Jana Třešňák Hercogová, Kristýna Sokolová

**Affiliations:** a Department of Dermatovenereology, Second Faculty of Medicine, Charles University, Bulovka University Hospital, Prague, Czech Republic; b Department of Pediatric Dermatology, Faculty Hospital and Masaryk University Brno, Czech Republic; c Department of Dermatovenereology, Third Faculty of Medicine, Charles University and University Hospital Kralovske Vinohrady, Prague, Czech Republic; d Department of Dermatology and Venereology, Faculty Hospital and Medical Faculty of Charles University, Hradec Králové, Czech Republic; e Department of Dermatology, Hospital Ceske Budejovice, Ceske Budejovice, Czech Republic; f Department of Dermatovenereology, Faculty of Medicine and University Hospital in Pilsen, Charles University, Pilsen, Czech Republic; g Department of Dermatology, Hercogova, Prague, Czech Republic

**Keywords:** adolescent, atopic dermatitis, effectiveness, lebrikizumab, safety

To the Editor:

Lebrikizumab, a novel anti-interleukin-13 antibody for atopic dermatitis (AD), has demonstrated efficacy and safety in clinical trials in adults and adolescents; however, real-world evidence in adolescents remains limited.^1^^,^^2^ This analysis evaluates the effectiveness and safety of lebrikizumab over 24 weeks in adolescents enrolled in the Czech BIOREP registry.

The study included 32 adolescent patients with moderate-to-severe AD who received an initial lebrikizumab dose from September 2024 to August 2025 (Table I). Of these, 30 patients (93.8%) attended at least 1 follow-up visit at week 16, and 25 (78.1%) completed the second follow-up at week 24. Only patients with baseline itch numeric rating scale (NRS) scores ≥4 (30/32) were evaluated for achieving an improvement of ≥4-point improvement. Throughout the study, patients continued to use moisturizers and various topical therapies as needed.

**Table I:** Baseline demographic characteristics and adverse events during the observed period

Characteristics	Lebrikizumab (*n* = 32)
Female, *n* (%)	17 (53.1%)
Age, mean (range), y	14 (12-17)
Disease duration, mean (range), y	12 (3-17)
BMI, mean (range)	20.8 (16.9-30.0)
Baseline EASI, mean (range)	28.8 (21.2-60.3)
Baseline Itch NRS, mean (range)	7.1 (2-10)
Baseline DLQI, men (range)	15.8 (1-26)
Atopic comorbidities, *n* (%)	
Food allergy	18 (56.3%)
Asthma	9 (28.1%)
Rhinitis	20 (62.5%)
Allergic conjunctivitis	0 (0.0%)
Prior systemic therapy, *n* (%)	
Phototherapy	32 (100.0%)
Cyclosporine	5 (15.6%)
Dupilumab	3 (9.4%)
Upadacitinib	2 (6.3%)
Lebrikizumab by line of targeted therapy, *n* (%)	
First line (naïve to biologics of JAK inhibitors)	27 (84.4%)
Second line	5 (15.6%)

Adverse events	Lebrikizumab (*n* = 32)
Any TEAE, *n* (%)	3 (9.4%)
Conjunctivitis	2 (6.3%)
Injection-site reaction	1 (3.1%)
Serious TEAE, *n* (%)	0 (0.0%)

*AD*, Atopic dermatitis; *BMI*, body mass index; *DLQI*, Dermatology Life Quality Index; *EASI*, eczema area and severity index; *NRS*, numeric rating scale; *TEAE*, treatment-emergent adverse event.

The mean eczema area and severity index (EASI) percentage change from baseline (28.8) was −77.4% (6.5) at week 16, improving slightly to −81.6% (5.3) by week 24. After week 16, 66.7% (20/30) of patients achieved an EASI-75 response and 43.3% (13/30) achieved an EASI-90 response. By week 24, 80.0% (20/25) of patients achieved an EASI-75 response, and 36.0% (9/25) achieved an EASI-90 response (Fig 1. Additional information in supplementary figure S1 available on Mendeley at https://data.mendeley.com/datasets/vgt9vjj99m/1). A >4-point reduction in Itch NRS was observed in 19/28 patients (67.9%) at week 16 and in 13/23 (56.5%) at week 24. The mean change in the Dermatology Life Quality Index from a baseline score of 15.8 was −10.1 at week 16 and −11.1 at week 24.

**Fig 1:**
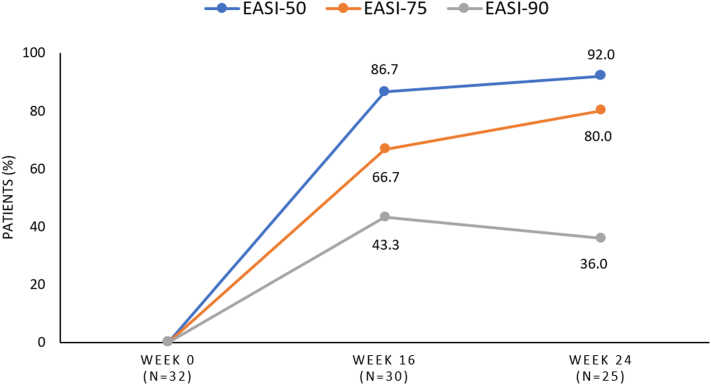
Clinical response after 16 and 24 weeks of lebrikizumab therapy in adolescents with atopic dermatitis.

At least one treatment-emergent adverse event was reported in 3 (9.7%) patients during the study period; none were considered serious. Conjunctivitis occurred in 2 patients (6.5%), and 1 patient (3.2%) experienced an injection-site reaction. During the observation period, 3 patients (9.4%) discontinued lebrikizumab after 24 weeks due to insufficient efficacy, while all remaining patients continued treatment.

The results of this study are consistent with findings from the pooled efficacy analysis of adolescents in the ADvocate1&2 trials, in which 62% of patients achieved an EASI-75 response and 41% achieved EASI-90 at week 16. Lebrikizumab also produced a substantial reduction in itch in real-world adolescent patients, with 53.3% achieving a ≥4-point improvement in Itch NRS, comparable with the pooled clinical trial analysis (48.9%).^1^ In line with clinical trial data, adolescents in this real-world cohort demonstrated marked improvements in quality of life, as measured by Dermatology Life Quality Index reduction at both 16 and 24 weeks.^1^^,^^2^

The safety profile of lebrikizumab observed in clinical trials involving adolescents is consistent with our findings; conjunctivitis occurred in 6.5% of patients in this cohort, comparable to the 5% reported by Paller et al.^2^ Based on clinical trial data and the limited real-world evidence available, conjunctivitis appears to be slightly less common in adolescents with AD than in adult patients treated with lebrikizumab.^2^, ^3^, ^4^, ^5^

Limitations of our study are the absence of data on topical therapies and the small number of patients.

In conclusion, this analysis, despite its limited sample size, supports the short-term effectiveness and safety of lebrikizumab in adolescents with AD in real-world clinical practice.

## Conflict of interest

Dr Rob has received consultancy and/or speaker honoraria from, and/or participated in clinical trials sponsored by, AbbVie, Almirall, Amgen, ArgenX, Boehringer Ingelheim, Bristol Myers Squibb, Celldex, Eli Lilly, Johnson & Johnson, LEO Pharma, MSD, Novartis, Pfizer, Sanofi, and UCB. Dr Pinková has received consultancy and/or speaker honoraria from, and/or participated in clinical trials sponsored by, AbbVie, Almirall, Pfizer, Sanofi, and LEO Pharma. Dr Gkalpakioti has received consultancy and/or speaker honoraria from, and/or participated in clinical trials sponsored by, AbbVie, Almirall, Eli Lilly, and Sanofi. Dr Bartoňová has nothing to disclose. Dr Horažďovský has received consultancy and/or speaker honoraria from, and/or participated in clinical trials sponsored by, AbbVie, Almirall, BMS, Eli Lilly, Johnson & Johnson, LEO Pharma, MSD, Novartis, Pfizer, Sanofi, and UCB. Dr Říčař has nothing to disclose. Dr Třešňák Hercogová has received consultancy and/or speaker honoraria from, and/or participated in clinical trials sponsored by, Almirall, Amgen, BMS, Bros Group, Fresenius Medical Care, Johnson & Johnson, and UCB, and is a co-owner of Dermatology Prof. Hercogova. Dr Sokolová has received consultancy and/or speaker honoraria from, and/or participated in clinical trials sponsored by, AbbVie, Almirall, Eli Lilly, Johnson & Johnson, Novartis, Pfizer, Sanofi, and UCB.

## References

[1] Hebert A.A., Flohr C., Hong H.C.H. (2024). Efficacy of lebrikizumab in adolescent patients with moderate-to-severe atopic dermatitis: 16-week results from three randomized phase 3 clinical trials. J Dermatolog Treat.

[2] Paller A.S., Flohr C., Eichenfield L.F. (2023). Safety and efficacy of lebrikizumab in adolescent patients with moderate-to-severe atopic dermatitis: a 52-week, open-label, phase 3 study. Dermatol Ther (Heidelb).

[3] Hagino T., Uchiyama A., Onda M. (Posted online February 20, 2025). Real-world effectiveness and safety of lebrikizumab for moderate-to-severe atopic dermatitis: a 16-week study in Japan. Dermatitis.

[4] Hagino T., Uchiyama A., Onda M. (Posted online march 18, 2025). 24-week real-world effectiveness and safety of lebrikizumab for atopic dermatitis in Japan: an analysis stratified by prior systemic therapy. Dermatitis.

[5] Rob F., Gkalpakiotis S., Kojanová M. (2025). Efficacy and safety of lebrikizumab in atopic dermatitis over 24 weeks: An analysis from the BIOREP registry. J Eur Acad Dermatol Venereol.

